# Development and Production of a Children’s Upper-Limb Cycling Adapter Using 3D Printing

**DOI:** 10.3390/ma17194731

**Published:** 2024-09-26

**Authors:** Barbora Kopová, Martin Bakeš, Martin Čížek, Adam Horký, Josef Dvořák, Karel Ráž, Zdeněk Chval

**Affiliations:** 1 Department of Industrial Engineering and Management, Faculty of Mechanical Engineering, University of West Bohemia, Univerzitní 2762/22, 301 00 Pilsen, Czech Republic; bakesm@students.zcu.cz; 2 Department of Machine Design, Faculty of Mechanical Engineering, Regional Technological Institute, University of West Bohemia, Univerzitní 2732/8, 301 00 Pilsen, Czech Republic; cizekmar@students.zcu.cz (M.Č.); dvorakj@kks.zcu.cz (J.D.); kraz@fst.zcu.cz (K.R.); zdchval@fst.zcu.cz (Z.C.); 3 Department of Design and Applied Arts, Ladislav Sutnar Faculty of Design and Art, University of West Bohemia, Univerzitní 2732/8, 301 00 Pilsen, Czech Republic; horkadam@students.zcu.cz

**Keywords:** upper-limb adapter, upper-limb prosthesis, bicycle adapter, children adapter, prototyping product design, 3D printed

## Abstract

The research described in this study focuses on the development of an innovative upper-limb adapter for young children aged 1–3 years who have congenital upper-limb defects. The objective was to create a functional and affordable solution that allows children to engage more safely and actively in physical activities such as cycling. The adapter was designed within the DESIGN+ project at the University of West Bohemia in Pilsen in collaboration with the German company Ottobock. The development included a detailed analysis of hand movements during cycling, modelling using CAD software (NX 1888), prototype manufacturing through 3D printing, and subsequent testing. The result is an adapter that allows 360° rotation around the arm axis, provides natural hand movement while turning, and is made of soft material to enhance safety. Despite initial challenges and necessary prototype adjustments, a functional and reliable design was achieved. This adapter will contribute to improving the quality of life for children with upper-limb disabilities, supporting their coordination, strength, and confidence in daily activities.

## 1. Introduction

An upper-limb defect represents a factor in a child’s life that significantly affects their motor function and quality of life. Most children with upper-limb defects are born with these conditions [1,2,3]. These defects include an increased number of fingers, a fusion of two or more fingers, an insufficient blood supply leading to amputation, and missing parts of the hand or an entire hand missing due to abnormal development during pregnancy [4,5]. The incidence of congenital upper-limb defects varies geographically, and complete records of such disabilities are rare in some countries. Studies indicate that the number of children born with upper-limb disabilities ranges from 4 to 30 per 10,000 births [4,6]. According to the Centers for Disease Control and Prevention (CDC), congenital upper-limb defects occur in 4.1 of every 10,000 live births in the United States. The European registry, EUROCAT, which collects data on congenital anomalies across Europe, reports an incidence of upper-limb defects ranging from 7 to 9 cases per 10,000 live births. There are slight variations between countries, with some reporting higher or lower rates. In Finland, a birth prevalence of 13 per 10,000 live births was found for the period 1964–1977, whereas in Scotland it was estimated at 30 per 10,000 live births for 1964–1968. Data from Australia show similar rates: approximately 4 out of 10,000 children [4,7,8,9]. Some studies suggest that the incidence of upper-limb defects may be slightly higher in male newborns compared to females. Differences between ethnic groups are generally minimal but may vary depending on access to healthcare, genetic factors, or cultural conditions [1,3,10]. 

Despite technological advancements, the majority of the children’s prosthetics market consists of cosmetic prostheses, which lack functionality. Young children commonly use and assist with their affected limb in manipulation, object exploration, crawling, or standing up [11]. Therefore, there should be an emphasis on developing specialized prostheses that enable the performance of activities that children with congenital defects cannot perform or can only perform with difficulty, such as cycling, gymnastics, and other sports [12,13,14]. An appropriately designed prosthesis should maximize the range of motion, ensure weight balance and stability, and provide comfort [15,16]. Besides limited availability, upper-limb prostheses for children are often not prescribed in a timely manner or are not affordable [15,17].

Properly timed prescription of the first prosthesis has a significant impact on the rate of acceptance among children. Studies confirm a higher likelihood of long-term prosthesis use if it is fitted before the child reaches two years of age. Introducing the prosthesis before the child’s first year resulted in its use for at least another four years. In contrast, prescribing a prosthesis after the second year led to an increased frequency of rejection [18,19,20]. A crucial factor in the acceptance of the prosthesis is its functionality. If the patient or the parent did not perceive a significant functional benefit from the prosthesis, interest in using it waned, leading to its rejection and to disappointment among parents [21].

Prosthetic options for children with upper-limb disabilities are limited. The availability of functional adapters expands only at a later age. Most patients and parents postpone acquiring specialized or advanced functional prostheses until adulthood due to their high cost. The necessary replacement of small prostheses to match the child’s development and growth multiplies the financial burden on families [22,23,24]. Prices of upper-limb prostheses vary and depend primarily on the type of prosthesis. The cosmetic prostheses can cost around €4500. For functional prostheses and adapters designed for sports, the price can go up to €10,000. The most expensive types are myoelectric, the purchase price of which ranges from €20,000 to €100,000. Beneficially, the development and production of more affordable functional attachments leads to a reduction in wear and tear or risk of damage to technologically complex and expensive myoelectric prostheses [25,26,27].

Advancements in 3D printing open up interesting possibilities in the field of prosthetics. Manufacturing using 3D printers is an ideal solution for transitional prostheses; 3D printing technology can significantly reduce previous problems related to cost, production speed, size changes, and small-series production [22,28,29]. Additionally, 3D printing enables the production of single-piece products without assembly needs. Prosthetic prototypes can be printed, tested, and improved, sometimes in a single day. Intriguing designs and the possibility of a wide range of color combinations are additional advantages, especially if the end-users are young children [30,31,32,33,34]. 

However, 3D printing is also associated with several concerns. Mechanical properties depend on the printing orientation, and their exact values are difficult to predict. Durability, sufficient grip strength, reproducibility, and shrinkage of the printed product call for further research or the development of new materials [35,36]. The authors of various studies address and analyze problems such as clogging of the nozzle in the FFF (Fused Filament Fabrication) 3D printing technique [37,38,39]. Limitations also arise during the printing of texts, patterns, and colors. Inkjet printing is often accompanied by the undesired formation of satellite droplets, which blur print patterns and contaminate both the printer and the surrounding environment. Numerous efforts have been made to mitigate or eliminate these droplets and to promote the generation of satellite-free drops [40,41].

The topic of 3D-printed prosthetics is a key and significantly researched area, with numerous relevant studies. Most scientific articles on pediatric prosthetics focus on the development and manufacture of 3D-printed upper-limb prostheses. These primarily involve functional prostheses that aim to replace missing limb parts, and allow for gripping light objects, bimanual manipulation, increasing the muscle strength and range of motion of the healthy upper limb, or facilitating the habit and control of a myoelectric prosthesis [42,43,44,45].

The research on 3D-printed prosthetics rarely addresses functional prostheses that would allow children with upper-limb disabilities to engage in sports and other activities, leading to an unrestricted life. One rare example is a study focused on prototyping and manufacturing a 3D-printed prosthetic hand that enables children to confidently participate in gymnastics classes and perform exercises on the horizontal bar [12].

Cycling is an integral part of childhood. Currently, the most accessible solutions for children without an upper limb are various bicycle attachments that are directly mounted on the handlebars. These are conceptually simple and are easily adaptable to the child’s hand. Unfortunately, the simplicity of the solution does not allow for natural duction movement while turning. Another concern is their comfort and safety in the event of a fall [46,47,48].

Adapters which are more complex and address the natural movement of the child while cycling are not commonly available on the market. Cycling prostheses or adapters for children are encountered only sporadically and are primarily specialized solutions developed for a specific young patient [49].

The aim of this study is to design a bicycle adapter for children. The main purpose of this product will be to improve the quality of life of young children who are born with an upper-limb defect or have lost a limb due to trauma or illness. With the adapter, children will engage the limb more actively and safely in physical activities, which will promote the development of their coordination and strength. Increasing children’s self-confidence, independence, and engagement in normal activities with peers is another motivation for the development of this upper-limb adapter. 

## 2. Materials and Methods

### 2.1. DESIGN+ Project

This study emerged from the innovative project “Design and Mechanical Engineering”. Known as DESIGN+, the project brings together students from various faculties of the University of West Bohemia in Pilsen, who collaborate to create technological innovations. Project assignments are created, consulted over, and subsequently evaluated by significant companies in Pilsen. Professor Stanislav Hosnedl from the Faculty of Mechanical Engineering of the University of West Bohemia has established and developed this project [50].

The sponsor for the DESIGN+ project for the year 2023/2024 was the German company Ottobock, which specializes in the manufacture and development of orthopedic and prosthetic aids for people with physical disabilities. Founded in 1919 in Berlin by the Bock family, Ottobock has become one of the world’s leading suppliers of prosthetic and orthopedic products. Ottobock provides innovative solutions that help people with limited mobility improve their quality of life. 

The company offers a wide range of products, including lower limb prostheses, upper-limb prostheses, orthoses, wheelchairs, and other orthopedic aids. These products are designed to simulate the movement of the human body as closely as possible, enabling users’ greater independence and mobility. Ottobock invests in research and development to continually improve its products and provide the most advanced technologies and designs for its customers. The company has a global presence, with branches worldwide, allowing it to help people in many countries. Ottobock is known for its commitment to the health and comfort of people with disabilities, and its products are often recommended by doctors and specialists in the field of orthopedics. The company also collaborates with various non-profit organizations and charitable institutions that support people with disabilities [51].

### 2.2. Main Goal and Target Group

The aim of the study was to develop an innovative upper-limb cycling adapter for young children aged 1–3 years who were born with an upper-limb defect or had to undergo amputation of part of their arm due to trauma or illness. The bicycle adapter was conceived, constructed, and designed to meet the specific needs of this target group, adhering to all specified and implicit requirements and standards. The task was to innovate the existing solutions in terms of concept, construction, and design. Key elements of the assignment included enabling 360° rotation of the adapter around the arm axis and solving the duction movement to prevent unnatural body rotation of the child while cycling. Another crucial requirement was to minimize production costs. For this reason, 3D printing was chosen as the manufacturing method.

Parents and caregivers play a crucial role in the process of introducing and using the adapter, and thus the product must be user-friendly and easily manageable. The active nature of children requires the adapter to be ergonomic and allow free movement while also being safe and impact resistant. Collaboration with experts in pedagogy and rehabilitation is key to successfully integrating the prosthesis into the child’s life. The prosthesis should be designed to facilitate the rehabilitation process and skill development in children.

### 2.3. Development Process Phases 

**Figure 1:**
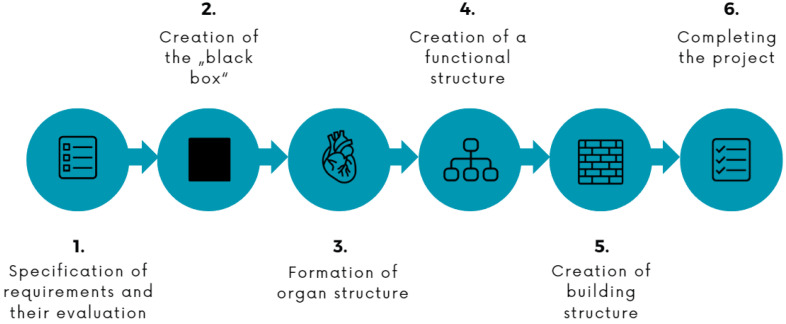
Workflow of the project’s process of creating a bicycle adapter for children.

The development of the project was approached systematically, as can be seen in Figure 1. First, the previously mentioned requirements for the adapter were specified and evaluated based on their importance and necessity. This was followed by the creation of a black-box diagram which displayed all the operators, inputs, and outputs forming the transformation process that enables a child with an upper-limb disability to ride a bike. The functional structure defined the necessary main functions of the adapter, such as enabling attachment of the adapter to a prosthesis, 360° rotation around the arm axis, smooth release of the adapter from the handlebars, duction movement, and enabling fixation of the rotation. The organ structure defined the types of components that met all the client’s requirements, and the functions were defined in the functional structure. The modelling of the adapter was divided into the gripping part, modelled using CAD software Rhinoceros, and the mechanism and connecting part, created in Siemens NX. During the project completion phase, the individual parts were assembled and prototypes were developed and improved. The final product was 3D printed, and the cycling adapter was evaluated with the children.

### 2.4. Architecture and Design Process

**Figure 2:**
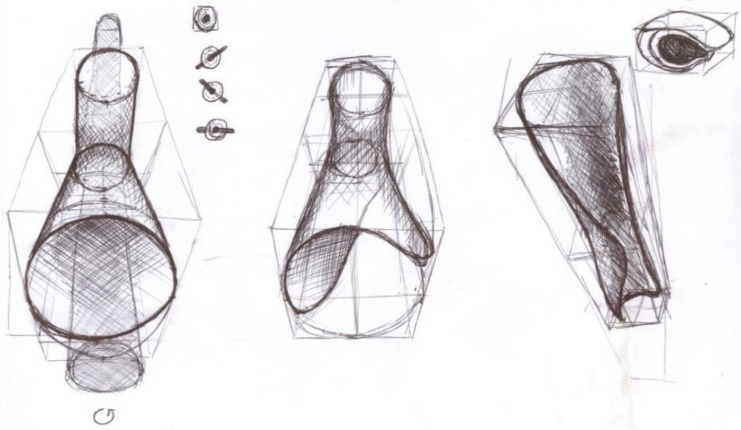
Sketches used to investigate the principle of hand movements in cycling.

This part of the study deals with a detailed description of the design process for the gripping part of the children’s adapter. At the beginning of the process, it was necessary to understand the principles of hand movements while riding a bike, focusing on the relationships between the movements of the forearm, wrist, hand, and handlebars. The main activities analysed in connection with cycling were gripping, holding, and releasing the handlebars (Figure 2). Furthermore, the movements associated with turning and tilting the hand while steering, as well as mechanisms for reducing shocks transmitted to the cyclist’s hands, were examined. Due to simplification and financial constraints, the analysis of movements associated with braking was excluded.

**Figure 3:**
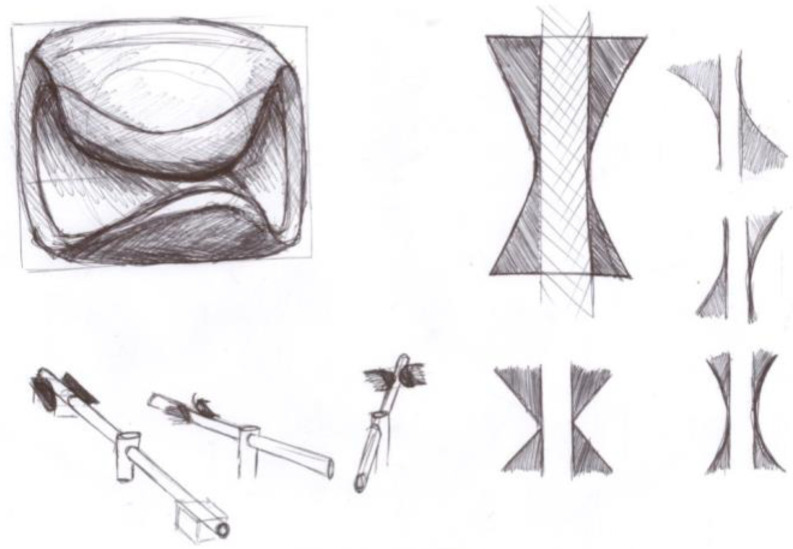
Designs of the hourglass shape of the gripping part of the adapter.

Based on a detailed analysis of hand behaviour while cycling, hand movements were transformed or simplified. Prototypes were designed using an hourglass-inspired shape (Figure 3), allowing natural turning and tilting of the gripping part when steering. A softer material was chosen to reduce shocks. The central axis of the prosthetic hand, designed with a narrower central part, ensures a firm grip on the handlebars and easy manipulation thanks to a special cut-out and flexible material.

**Figure 4:**
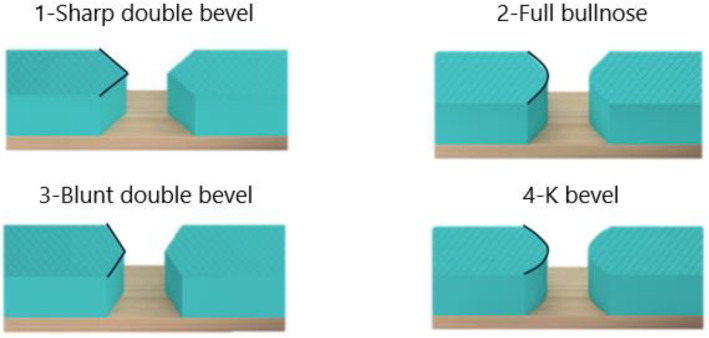
Different shapes of the wood and polystyrene test models.

Designs utilizing available materials and simple manufacturing processes were created as simplified verification models made of wood, polystyrene, and adhesive tape (Figure 4). They were produced on an adult scale and subjected to testing, which included evaluating various shapes, bevel angles, and sizes. The main factors in the testing were the quality of the grip on the handlebars and the ease of turning. Option 2 was chosen for the final design of the adapter. The slight chamfering and the bulkhead allowed for a natural and controlled movement of the handlebars when cornering.

**Figure 5:**
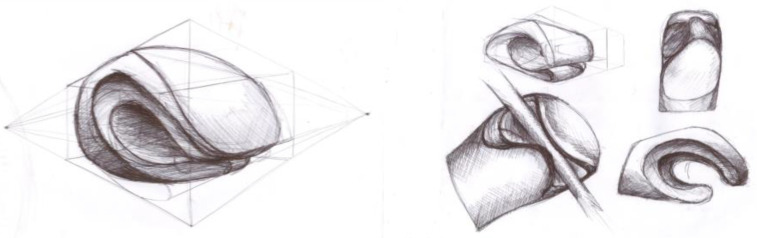
Sketches of the final shape of the adapter.

After completing the functional part of the prosthetic hand, the next phase was the development of the outer shell. A set of conditions was established for the design of the outer shell, which had to meet the following requirements: child safety, ease of printing, sufficient strength under normal load and wear, maintainability, adaptability to the child’s size, compatibility with the child’s balance bike and corresponding adapter, and an attractive, toy-like appearance for children. Based on these conditions, a basic version of the final product was designed and created (Figure 5). 

**Figure 6:**
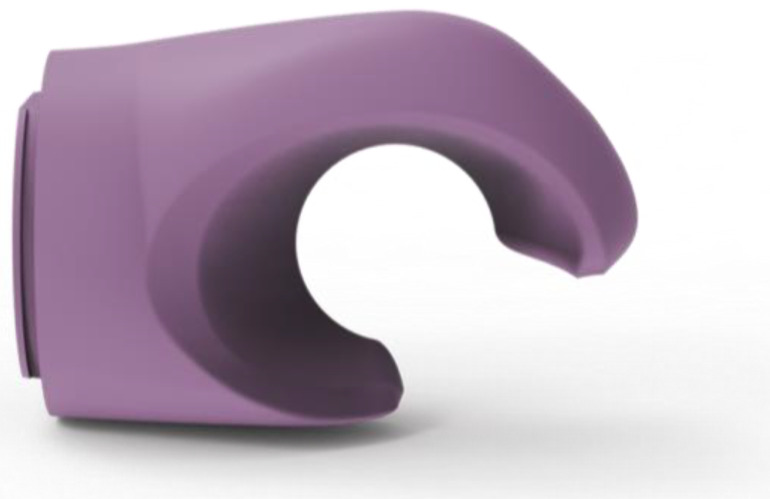
The 3D model of the gripping part of the adapter.

The prototype in Figure 6 was prepared for further modifications and testing. Initial tests and individual feedback from children will allow for further optimization of the design, leading to a final product that is safe, functional, and attractive.

### 2.5. Development of the Adapter Mechanism

Several concepts were developed for the mechanism. The focus during the development was primarily on simplicity due to the chosen production method of 3D printing. For both the printing and subsequent assembly, it holds true that the more parts there are, the more expensive the concept becomes. Flexion is partly allowed by the rotation of the prosthesis’s clamping part, which also permits another movement—duction. The main function that the mechanism must fulfill is enabling 360° rotation of the adapter and its disconnection/connection while stationary. To facilitate future development, it was necessary to enable the detachment of the adapter’s gripping part so that a different attachment for another activity or sport could be connected to the universal mechanism.

**Figure 7:**
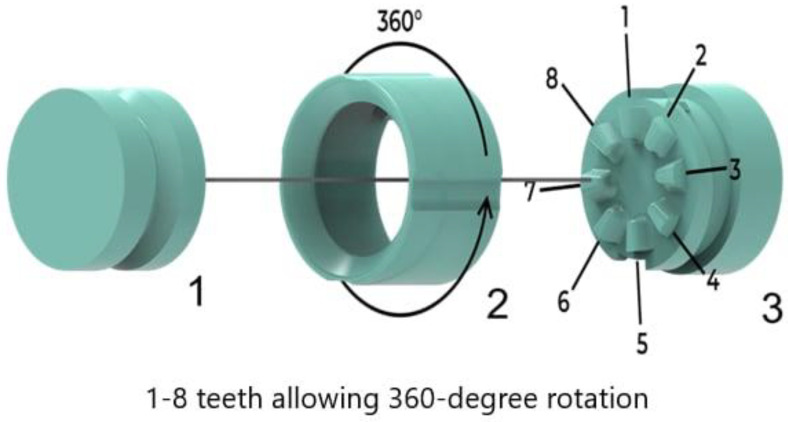
Functional parts of the mechanism (1—part that is connected to the prosthetic, 2—nut, and 3—bed part).

The mechanism consists of three parts, which can be seen in Figure 7 (1—the part that is connected to the prosthetic, 2—the nut, and 3—the bed part). Rotation around the axis to 360° is ensured by a toothed mechanism with eight shaped teeth, and the fixation of individual positions is enabled by a nut on the outer side of the adapter. By tightening the nut, the teeth of the toothed mechanism are fixed, making the adapter firm, stable, and sufficiently safe for riding a balance bike.

The prototypes were produced using the FDM (Fused Deposition Modeling) 3D printing method. The principle of the method is the gradual deposition of molten material in layers. The advantages of FDM include its availability, ease of use, low cost, speed of production, and low waste production. These are the main reasons why FDM 3D printing is the ideal choice for prototyping, visualizing, and creating functional parts [52,53,54]. In particular, the Original Prusa MK4 3D printer and polylactic acid (PLA) filament were used for the prototyping of the mechanism’s parts. PLA is a fully biodegradable material made from corn, potato starch, or sugar cane, and its use is environmentally friendly [55,56,57,58]. 

In the creation of the first functional prototype, several serious functional issues were discovered. Firstly, the nut (i.e., the positioning and connecting mechanism) did not work as intended. The functional surface of the thread, where the nut and the socket part of the adapter come into contact, lacked sufficient smoothness due to poor 3D printing quality, which impaired the thread’s functionality. Specifically, unwanted supports created on this surface during the printing process needed to be removed. While this issue was caused by the 3D printing technology, specifically FDM, it was essential to address this problem for future prototypes. Another complication was excessive clearance between the adapter’s components. Although clearance itself might not be a major problem, it was not accounted for in the thread design (i.e., thread pitch, and overall thread height). Consequently, during nut-tightening, the nut spun because the thread was too shallow. The final issue was the manufacturing of the combined nut-and-socket part. For simplicity, these two parts were designed to be printed as rotationally connected, reducing the problem of joining them. However, it was found that such parts could not be manufactured using FDM technology, necessitating adjustments to this joint.

For the second prototype, several changes were made. The previously mentioned rotational joint of the nut and the socket part was retained, but its shape was modified. Since the FDM technology can only produce steep parts (for parts without any underlying material) up to a 45° angle without supports, a V-shaped joint with a 90° apex angle (resulting in the desired 45° residual angles) was used. This 45° chamfer was also applied to the thread, eliminating the issue of the screw surface’s roughness. Finally, a method was developed to connect the gripping part with the rest of the prosthesis. This connection was intended to be achieved through a shape fit. While this prototype appeared to successfully address all functional problems, it became apparent that play between the nut and the socket part, along with manufacturing inaccuracies, continued to cause the nut to spin and prevent proper connection.

**Figure 8:**
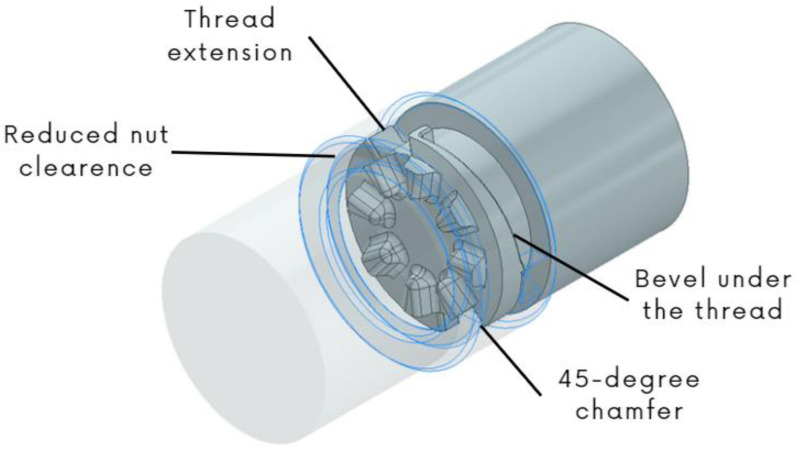
Modified prototype of the mechanism.

In the third and final prototype, the thread design accounted for play. The thread was designed with the maximum clearance allowance, ensuring with a certain degree of safety that the nut would not spin. Additionally, several minor modifications were made. Grooves were added to the nut to facilitate tightening and loosening the connection and to improve the overall handling of the adapter. The groove for attaching the adapter’s gripping part was also adjusted. After manufacturing this prototype, the adapter functioned as intended. The final prototype of the adapter mechanism with these changes can be seen in Figure 8.

Finally, one more change was made, one which did not require further testing. This involved grooves for laminating the socket part, purely for attaching the laminate to the rest of the prosthesis. With this, the product was completed.

## 3. Results

### 3.1. Final Product

This section will briefly describe the functional features of the final product. Firstly, it is important to mention that the current version of the product, specifically this adapter, is primarily designed for use on balance bikes for children aged 1 to 3 years. When riding a balance bike, it is crucial to ensure a secure and reliable connection between the prosthesis and the handlebars, and to enable radial and ulnar deviation (side movement of the hand at the wrist), which is facilitated by the shape of the adapter’s gripping part. During turns, the position of the hands relative to the rest of the upper limbs changes. For a prosthetic arm, this movement must be compensated for by laterally rotating the entire adapter, which is firmly connected to the rest of the prosthesis, relative to the handlebars of the balance bike. The connection between the prosthesis and the handlebars is secured by a shape-expanding flexible clamping part. Its shape wraps around the circular profile of the handlebars, and to detach the adapter, it is necessary to forcibly expand the clamping part again.

Another feature offered by the final product is the rotational adjustment of the adapter’s position. There are a total of eight unique positions available. The positioning and connecting mechanism, consisting of a nut and two interlocking parts with complementary shape projections, facilitates the position adjustment. When the nut is loosened, the adapter can be freely rotated; however, this state is intended only for manipulation at rest. Despite this, the mechanism includes a shape lock that prevents accidental disconnection of the adapter from the rest of the prosthesis during manipulation. Thus, even when the connection is loosened during use, the adapter will not disconnect but will only rotate relative to itself.

**Figure 9:**
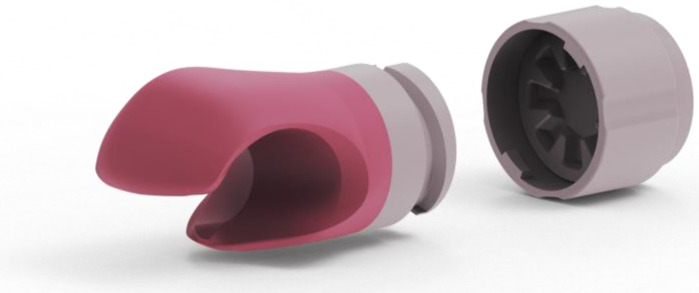
Final model of the adapter with mechanism.

The entire product, which is shown in Figure 9, is designed to be manufacturable using 3D printing technology. This allows it to be produced at a relatively low cost compared to prostheses manufactured in small quantities using other technologies.

### 3.2. The 3D Printing Production of the Adapter

There are many different methods of 3D printing. While the prototypes were produced using the FDM (Fused Deposition Modeling) method, which resulted in problems with the supports created and the subsequent poor surface quality, the final product was produced using the MJF (Multi Jet Fusion) method [59,60,61,62,63]. MJF is a method which, together with the widely used SLS (Selective Laser Sintering) method, belongs to the Powder Bed Fusion group of methods, techniques in which the powder is sintered. In MJF, unlike SLS, a laser is not used, but infrared radiation and an auxiliary agent are utilized instead [62,64,65,66].

**Figure 10:**
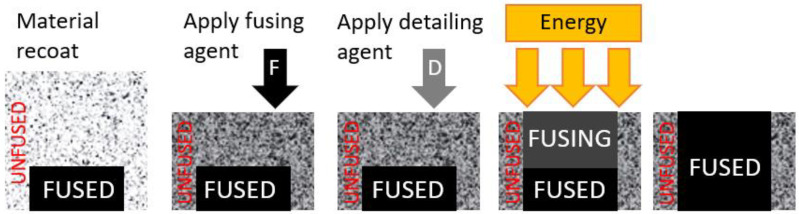
The principle of MJF technology [44].

In the production of a product using the MJF method, a layer of powder (material) is first applied to the working surface. Then, a black Fusion Agent is applied to certain areas to be fused and another agent (the Detailing Agent) is applied to areas close to that part. Infrared heaters are then run over the production area to sinter the areas primarily colored with the black Fusion Agent. Once the printed layer has settled, another batch of powder is applied, and the process is repeated until the product is finished. The whole process is illustrated graphically in Figure 10 [64,67,68]. 

The final product was manufactured using the MJF method and the PA12GB material. The thickness of one layer of PA12GB material is approximately 0.08 mm, resulting in a productivity of up to 4115 cm^3^ per hour. This material is processed at temperatures between 160 °C and 180 °C, which must be taken into account due to its melting point of 186 °C. [66,69,70]. According to official data, PA12GB has a tensile strength of 30 MPa and a guaranteed breaking elongation of 6.5% [71].

### 3.3. Testing and Validation

**Figure 11:**
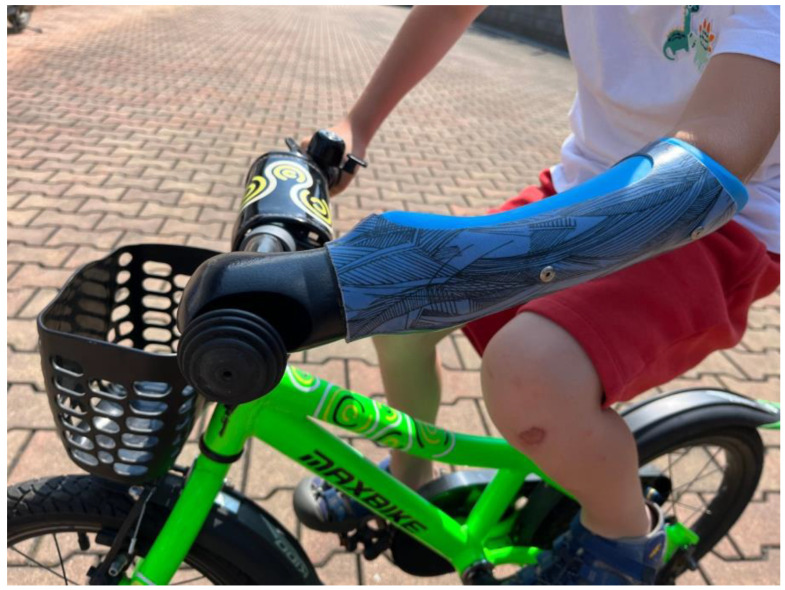
Testing a child adapter fitted to a prosthesis while cycling.

The evaluation was conducted using a version without the wrist mechanism, as illustrated in Figure 11. Consequently, this component was excluded from the assessment, and no conclusions can be drawn regarding its performance. The focus of the evaluation was therefore directed toward the functionality of the palm itself. 

The cycling test with the attachment was conducted by three children, two of whom were 5 years old, and one 4 years old. The testing aimed to assess the usability and effectiveness of the attachment in a real-world scenario, providing insights into its suitability for young users. The children’s overall response was predominantly positive. Initially, the first examiner expressed uncertainty regarding the motion of the duction, which was controlled by the hour-glass shape. However, after a few minutes of adaptation, the cycling with the attachment was mastered successfully. For the youngest participant, aged 4, the bicycle attachment was relatively large and required resizing to ensure a proper fit.

From a functional point of view, the palm proved its worth. It held steady on the handlebars, which is a positive finding. However, the material could have been made a little softer by reducing the percentage of padding when printing. A softer palm would result in increased flexibility and ease of fitting on the handlebars. One promising avenue involves exploring the use of nanomaterials, which could offer enhanced flexibility and durability, in addition to being lightweight. Nanomaterials can be engineered to provide superior cushioning while maintaining strength, potentially leading to significant advancements in user comfort and performance [72,73,74].

The patient initially showed uncertainty in moving the palm, but gradually became familiar with the movement. Eventually, he was pleasantly surprised by the palm movement and seemed to find it beneficial. 

Testing on a larger sample of users would be useful in order to obtain more relevant data on the functionality and comfort of the palm movement. However, this is currently not possible due to time limits and financial constraints. For the next stages of development, it is crucial to optimize the filler settings. This would allow us to better respond to the needs of the users and adapt the functioning of the palm to their specific requirements.

## 4. Discussion

The results of this study indicate that the designed upper-limb adapter for young children can significantly contribute to their ability to engage in physical activities such as cycling. This project builds upon previous research focused on developing prosthetic aids for children with congenital defects. Unlike earlier studies that often addressed only static or limited-mobility prosthetics, our adapter allows full 360° rotation and natural duction movement during cycling.

The interpretation of the results, given the perspectives of previous studies, suggests that the integration of soft materials and ergonomic design can significantly enhance the comfort and safety of using adapters. This approach was supported by our findings, as children testing the adapter demonstrated improved coordination and greater confidence while cycling.

The potential implementations of these results are broad in nature. The adapter not only enables children with upper-limb disabilities to participate more actively in physical activities but also supports their psychomotor development, improves their physical condition, and enhances social integration. This can result in long-term positive impacts on their quality of life. 

Despite the positive outcomes, the study highlights areas for further improvement. Feedback from testing indicated that, while the palm demonstrated adequate functionality, the use of a softer material could enhance both comfort and fit. This suggests the potential for material optimization, which warrants further investigation. Future research may explore the application of nanomaterials to achieve these properties, potentially leading to improved overall performance. Additionally, conducting tests on a larger sample size would yield more robust data regarding the adapter’s functionality and comfort, thereby providing more comprehensive insights. 

Future research should include further design improvements based on user feedback and clinical trials. It would also be beneficial to expand the research to a larger sample of children and monitor their progress and experiences with adapting to the new tool over time. Another step could be the development of additional types of adapters for various sports and physical activities, which would further expand opportunities for children with upper-limb disabilities.

This study provides important insights and advancements in the field of adaptive aids for children with upper-limb disabilities and demonstrates that innovative design and modern technologies, such as 3D printing, can significantly contribute to improving their living conditions.

## 5. Conclusions

Based on the results of this study, it was possible to design and develop an innovative upper-limb adapter for young children aged 1–3 years who have congenital upper-limb defects. This adapter was conceived with the specific needs of this target group in mind and brings several key improvements over existing solutions.

The primary goal of developing this adapter was to enhance the quality of life for young children by enabling them to participate more safely and actively in physical activities such as cycling. Throughout the development process, detailed analyses of hand movements during cycling were conducted, followed by CAD modeling, prototype manufacturing via 3D printing, and subsequent testing with children. The result is an adapter that allows 360° rotation around the arm axis, provides natural hand movement during turning (ulnar deviation), and is made from a soft material to enhance safety.

Despite initial challenges and necessary prototype adjustments, a functional and reliable design was achieved. The adapter was tested and optimized based on feedback from children and their parents, leading to a final product that meets all required functional and safety standards.

A key advantage of using 3D printing technology in the development of this adapter is the ability to quickly produce and modify prototypes. Utilizing 3D printing not only reduces production costs but also shortens the time required for product development and optimization. Moreover, it offers the possibility of customizing the product to meet the specific needs of individual users, which is particularly important for young children, who grow rapidly and require frequent adjustments to their prosthetic aids.

The DESIGN+ project at the University of West Bohemia in Pilsen, in collaboration with the German company Ottobock, demonstrated how interdisciplinary collaboration can lead to innovative and practical solutions. The use of 3D printing played a crucial role in rapid prototyping and testing, allowing for efficient design iteration and improvement of the final product.

The resulting adapter for children with upper-limb disabilities has the potential to significantly improve their ability to engage in everyday activities with peers, thereby supporting their physical and psychological development. The adapter not only enhances their coordination and strength but also contributes to their self-confidence and independence. 

This project not only provides a functional solution for children with upper-limb disabilities but also opens doors for further research and development in the field of pediatric prosthetics. The results of this study can serve as a foundation for future projects focused on developing additional prosthetic aids and adapters, which could further improve the living conditions and opportunities for children with disabilities.

Overall, this project represents a significant step forward in the field of pediatric prosthetics and demonstrates that through modern technologies, such as 3D printing, substantial advancements can be achieved in improving the lives of children with disabilities.

## Data Availability

The original contributions presented in the study are included in the article, further inquiries can be directed to the corresponding author/s.
